# Recurrent Endometrial Stromal Sarcoma: Treatment with a Progestin and Gonadotropin Releasing Hormone Agonist

**DOI:** 10.1155/2010/353679

**Published:** 2010-06-10

**Authors:** Nefertiti Chianti duPont, Philip John DiSaia

**Affiliations:** Division of Gynecologic Oncology, Department of Obstetrics and Gynecology, University of California, Irvine College of Medicine, Orange, CA 92868, USA

## Abstract

Endometrial stromal sarcoma (ESS) formerly classified as low-grade endometrial stromal sarcoma is a rare uterine malignancy with a good prognosis despite a tendency to recur. Primary surgical management for ESS includes total abdominal hysterectomy and bilateral salpingo-oophorectomy. Patients with ESS have long disease-free survival rates when treated with primary surgical therapy, but nearly fifty percent of these patients will recur. We present the case of a patient with recurrent ESS who had an excellent response to combined therapy with megestrol and leuprolide.

## 1. Introduction

First reported in 1908, endometrial stromal sarcoma (ESS) is a rare uterine tumor known for its slow growth [1]. Originally known as interstitial endometrioma, endolymphatic stromal myosis, or low-grade endometrial stromal sarcoma ESS is recognized by its infiltrating margins and low mitotic activity [2–4]. Endometrial stromal sarcomas represent 0.2% of all genital tract malignancies and were previously subdivided into low- or high-grade categories based upon their mitotic index. High-grade endometrial stromal sarcomas are now termed poorly differentiated or undifferentiated uterine sarcomas due to its aggressive clinical course [5–10]. 

Typically diagnosed postoperatively on hysterectomy specimens, ESS has an indolent course and recurrent disease can occur up to twenty years after diagnosis. Primary ESS is treated surgically with a total abdominal hysterectomy and bilateral salpingo-oophorectomy and routine lymphadenectomy has not been found to improve overall survival rates [1, 3, 7, 8]. Recurrent ESS is commonly treated with progestins like other endometrial cancers but there is no consensus regarding optimal management [5]. ESS is hormonally responsive and contains estrogen and progesterone receptors [5, 11–13]. Patients with retained ovaries after a hysterectomy or those who receive estrogen hormone replacement have high recurrence rates; therefore, single agent estrogen replacement therapy is not recommended [5]. Progestins however are commonly used in the adjuvant setting to treat ESS [5]. The mechanism of action of progestins is to bind progesterone receptors and cause downregulation of gene transcription leading to decreased endometrial gland and stromal proliferation [11, 14]. Diminished endometrial gland and stromal proliferation make progestins beneficial for use in the adjuvant setting and at the time of disease recurrence. In addition, gonadotropin releasing hormone (GnRH) agonists downregulate GnRH receptors in the anterior pituitary leading to a hypoestrogenic state. Here, we present the long-term followup of a patient with recurrent endometrial stromal sarcoma treated with a progestin and GnRH agonist with excellent results.

## 2. Case Report

A 41-year-old woman gravida 2 para 1 with a body mass index of 25 presented with menorrhagia, pelvic pain, and dysmenorrhea. Her past medical history was unremarkable. Her past surgical history was pertinent for a bilateral tubal ligation in 1991. After examination by her general gynecologist, she was found to have an enlarged uterus. No additional preoperative testing was performed. In November 1996, she underwent a total abdominal hysterectomy with bilateral salpingo-oophorectomy. The frozen section diagnosis revealed a uterine stromal tumor with rare mitotic figures. The final pathology diagnosed an International Federation of Gynecology and Obstetrics (FIGO) stage IA endometrial stromal sarcoma extending to the outer one third of the myometrium [15]. The fallopian tubes, ovaries, and uterine serosa were negative for malignancy. The uterus weighed 647 grams and measured 12.9 × 12 × 7 cm. Her postoperative course was uneventful and she required no further therapy. She continued her followup care under the management of her general gynecologist and remained without evidence of disease for two months.

 A surveillance computer tomography of the abdomen and pelvis performed in January 1997 showed three 4-5 mm nodular densities in the right lower lobe of the lung. The densities were too small to biopsy and no comparison films were available. The patient was asymptomatic and therefore was managed conservatively. The lung densities remained stable in size until August 1997 when one density was found to have increased to 15 × 14 mm. A lung biopsy of the largest lesion was suspicious, but not diagnostic for a malignancy. The enlarged lung density was thought to clinically represent recurrent disease and the patient was referred to a medical oncologist in her area who recommended cytotoxic chemotherapy. The patient declined chemotherapy and desired a second opinion. 

After referral to our medical center in November 1997, she was started on a regimen of megestrol 40 mg twice daily with monthly intramuscular injections of leuprolide 7.5 mg. Complete resolution of her lung nodules occurred in response to this regimen by January 1998. She remained on megestrol and a 3.75 mg dose of leuprolide until the date of last followup in June 2006. Now over 10 years after her initial surgery, she remains without evidence of disease.

## 3. Discussion

Due to the rarity of ESS, it is difficult to conduct prospective randomized clinical trials to determine the optimal treatment regimen. Treatment has been defined by the experience gained from retrospective case series and case reports. The patient in this case was treated with megestrol and leuprolide based on our clinical experience with this regimen. Our experience with this combination has influenced the use of progestins with GnRH agonists by graduates of our department [16]. 

Treatment of recurrent ESS is unclear. Chu and colleagues published findings showing that 75% of patients with stage I disease did not recur if treated with adjuvant megestrol compared to 29% of similarly staged patients who did not receive adjuvant megestrol [5]. Based on these findings, the authors recommend adjuvant megestrol 160 mg daily [5]. Several other studies have highlighted the use of GnRH agonists, progestins, and aromatase inhibitors alone or in combination with effective therapies in the treatment of ESS [17]. One case report published in 2004 showed good response with single agent GnRH analogue, triptorelin, in a patient with recurrent ESS [18]. Recurrent ESS has also been treated with radiation, surgical reexcision, chemotherapy, or a combination of these modalities, however, data supporting these treatments are limited [5, 11, 19–24]. Hormonal therapy has been studied the most in ESS. In one case series of 13 women with recurrent ESS who received hormonal management, 46% had an objective response [21]. The type of progestin varied in this study, but despite the type of progestin used (megestrol, medroxyprogesterone acetate, or hydroxyprogesterone acetate), all of the patients treated with hormonal management had favorable outcomes, leading us to believe that the type of progestin is not as important as the effect of progestins on this disease entity [21]. While several other studies have documented the effectiveness of other hormonal agents such as tamoxifen, letrozole, or amnioglutethimide for the treatment of recurrent ESS, progestins are inexpensive, have a favorable safety profile, and have been extensively studied in the treatment of uterine malignancies [17, 20, 24–26]. Although small and retrospective in nature, these studies illustrate the importance of the hypoestrogenic environment provided by progestins and GnRH agonists in the management of recurrent ESS.

In conclusion, ESS is a rare uterine malignancy with long disease-free intervals. ESS is rarely diagnosed preoperatively and many patients will develop disease recurrence. We have had success in the long-term management of one patient with recurrent ESS using megestrol and leuprolide and we recommend its use but multicenter prospective trials are needed to determine optimal therapy for this disease entity in the recurrent setting.

## References

[1] Doran A, Lockyer C (1908). Two cases of uterine fibroids showing peritheliomatous changes; long immunity from recurrence after operation. *Proceedings of the Royal Society of Medicine*.

[2] Goodall JR (1940). Endometrioma interstitiale: a preliminary report. *The Journal of Obstetrics and Gynaecology of the British Empire*.

[3] Berchuck A, Rubin SC, Hoskins WJ, Saigo PE, Pierce VK, Lewis JL (1990). Treatment of endometrial stromal tumors. *Gynecologic Oncology*.

[4] Norris HJ, Taylor HB (1966). Mesenchymal tumors of the uterus. I. A clinical and pathological study of 53 endometrial stromal tumors. *Cancer*.

[5] Chu MC, Mor G, Lim C, Zheng W, Parkash V, Schwartz PE (2003). Low-grade endometrial stromal sarcoma: hormonal aspects. *Gynecologic Oncology*.

[6] Kempson RL, Hendrickson MR (2000). Smooth muscle, endometrial stromal, and mixed Müllerian tumors of the uterus. *Modern Pathology*.

[7] Amant F, Moerman P, Cadron I, Neven P, Berteloot P, Vergote I (2003). The diagnostic problem of endometrial stromal sarcoma: report on six cases. *Gynecologic Oncology*.

[8] Riopel J, Plante M, Renaud M-C, Roy M, Têtu B (2005). Lymph node metastases in low-grade endometrial stromal sarcoma. *Gynecologic Oncology*.

[9] Chan JK, Kawar NM, Shin JY (2008). Endometrial stromal sarcoma: a population-based analysis. *British Journal of Cancer*.

[10] Leath CA, Huh WK, Hyde J (2007). A multi-institutional review of outcomes of endometrial stromal sarcoma. *Gynecologic Oncology*.

[11] Katz L, Merino MJ, Sakamoto H, Schwartz PE (1987). Endometrial stromal sarcoma: a clinicopathologic study of 11 cases with determination of estrogen and progestin receptor levels in three tumors. *Gynecologic Oncology*.

[12] Lantta M, Kahanpää K, Kärkkäinen J, Lehtovirta P, Wahlström T, Widholm O (1984). Estradiol and progesterone receptors in two cases of endometrial stromal sarcoma. *Gynecologic Oncology*.

[13] Zhu X-Q, Shi Y-F, Cheng X-D, Zhao C-L, Wu Y-Z (2004). Immunohistochemical markers in differential diagnosis of endometrial stromal sarcoma and cellular leiomyoma. *Gynecologic Oncology*.

[14] Giangrande PH, Kimbrel EA, Edwards DP, McDonnell DP (2000). The opposing transcriptional activities of the two isoforms of the human progesterone receptor are due to differential cofactor binding. *Molecular and Cellular Biology*.

[15] Mutch DG (2009). The new FIGO staging system for cancers of the vulva, cervix, endometrium and sarcomas. *Gynecologic Oncology*.

[16] Scribner DR, Walker JL (1998). Low-grade endometrial stromal sarcoma preoperative treatment with Depo-Lupron and Megace. *Gynecologic Oncology*.

[17] Leunen M, Breugelmans M, De Sutter Ph, Bourgain C, Amy JJ (2004). Low-grade endometrial stromal sarcoma treated with the aromatase inhibitor letrozole. *Gynecologic Oncology*.

[18] Burke C, Hickey K (2004). Treatment of endometrial stromal sarcoma with a gonadotropin-releasing hormone analogue. *Obstetrics and Gynecology*.

[19] Mansi JL, Ramachandra S, Wiltshaw E, Fisher C (1990). Endometrial stromal sarcomas. *Gynecologic Oncology*.

[20] Spano J-P, Soria J-C, Kambouchner M (2003). Long-term survival of patients given hormonal therapy for metastatic endometrial stromal sarcoma. *Medical Oncology*.

[21] Piver MS, Rutledge FN, Copeland L, Webster K, Blumenson L, Suh O (1984). Uterine endolymphatic stromal myosis: a collaborative study. *Obstetrics and Gynecology*.

[22] Matsuura Y, Yasunaga K, Kuroki H, Inagaki H, Kashimura M (2004). Low-grade endometrial stromal sarcoma recurring with multiple bone and lung metastases: report of a case. *Gynecologic Oncology*.

[23] Yokoyama Y, Ono Y, Sakamoto T, Fukuda I, Mizunuma H (2004). Asymptomatic intracardiac metastasis from a low-grade endometrial stromal sarcoma with successful surgical resection. *Gynecologic Oncology*.

[24] Maluf FC, Sabbatini P, Schwartz L, Xia J, Aghajanian C (2001). Endometrial stromal sarcoma: objective response to letrozole. *Gynecologic Oncology*.

[25] Dombernowsky P, Smith I, Falkson G (1998). Letrozole, a new oral aromatase inhibitor for advanced breast cancer: double-blind randomized trial showing a dose effect and improved efficacy and tolerability compared with megestrol acetate. *Journal of Clinical Oncology*.

[26] Amant F, Coosemans A, Debiec-Rychter M, Timmerman D, Vergote I (2009). Clinical management of uterine sarcomas. *The Lancet Oncology*.

